# A Deep Learning-Based Chinese Semantic Parser for the Almond Virtual Assistant

**DOI:** 10.3390/s22051891

**Published:** 2022-02-28

**Authors:** Shih-wei Liao, Cheng-Han Hsu, Jeng-Wei Lin, Yi-Ting Wu, Fang-Yie Leu

**Affiliations:** 1 Department of Computer Science and Information Engineering, National Taiwan University, Taipei 10617, Taiwan; liao@csie.ntu.edu.tw (S.-w.L.); gogameboy11@gmail.com (C.-H.H.); 2 Department of Information Management, Tunghai University, Taichung 407224, Taiwan; a0927313523@gmail.com; 3 Department of Computer Science, Tunghai University, Taichung 407224, Taiwan

**Keywords:** semantic parsing, Genie, Chinese, deep learning, multiple question answer network, ThingTalk, virtual assistant

## Abstract

Almond is an extendible open-source virtual assistant designed to help people access Internet services and IoT (Internet of Things) devices. Both are referred to as skills here. Service providers can easily enable their devices for Almond by defining proper APIs (Application Programming Interfaces) for ThingTalk in Thingpedia. ThingTalk is a virtual assistant programming language, and Thingpedia is an application encyclopedia. Almond uses a large neural network to translate user commands in natural language into ThingTalk programs. To obtain enough data for the training of the neural network, Genie was developed to synthesize pairs of user commands and corresponding ThingTalk programs based on a natural language template approach. In this work, we extended Genie to support Chinese. For 107 devices and 261 functions registered in Thingpedia, 649 Chinese primitive templates and 292 Chinese construct templates were analyzed and developed. Two models, seq2seq (sequence-to-sequence) and MQAN (multiple question answer network), were trained to translate user commands in Chinese into ThingTalk programs. Both models were evaluated, and the experiment results showed that MQAN outperformed seq2seq. The exact match, BLEU, and F1 token accuracy of MQAN were 0.7, 0.82, and 0.88, respectively. As a result, users could use Chinese in Almond to access Internet services and IoT devices registered in Thingpedia.

## 1. Introduction

Internet services are becoming more and more mature, and fast evolving ICT (Information and Communication Technologies) consistently enhance our user experiences. Today, people can use smartphones to not only access various Internet services, such as news websites and social networks, but also to control smart appliances in the home, such as air conditioners, TVs, refrigerators, and so on. It was estimated that the number of online IoT (Internet of Things) devices was more than 27 billion in 2016 [1]. With help from virtual assistants, people can use natural language to access Internet services and control IoT devices. By simply saying a command to a smartphone, such as “get a picture of cat,” people expect that virtual assistants will understand the command, process the request, and finally respond to the user.

Virtual assistants have been researched and developed in many domains, such as education [2], health care [3], entertainment [4,5], etc. At the time this paper was written, most virtual assistants were closed, i.e., designed and developed for only a specific use case, and thus not extendable by others. Dixon et al. proposed HomeOS, an operating system for smart devices in the home, and a set of interfaces for them [6]. Gordon and Breazeal proposed a car entertainment assistant [4]. It aimed to help drivers interact with their children in the back seats and prevent them from distraction. It was designed specifically for children’s entertainment in a car. Several giant companies have developed IoT assistants for their own smart devices and cloud services, such as Amazon Alexa [7,8], Apple Homekit [9], OpenWeave (Google Weave previously) [10], and Samsung SmartThings [11]. Usually, users have to use multiple virtual assistants to access different services. It is not uncommon that virtual assistants have compatible issues. Amazon Alexa, based on the Alexa Meaning Representation Language (AMRL) [8], can integrate third-party devices. It works with an ontology of about 20 manually tuned domains. This is very labor-intensive. It is not a simple task for ordinary users to extend the ability of these virtual assistants.

Almond [12,13,14] is an open-source virtual assistant that takes several important issues into consideration, such as privacy, extensibility, and programmability. It can execute codes written in ThingTalk, a high-level declarative domain-specific programming language designed to access Internet services and IoT devices that are both referred to as skills in this article. It uses a semantic parser to translate user commands in natural language into ThingTalk programs. The parser is a very large artificial neural network (ANN). To train such a network, a large amount of user commands and corresponding ThingTalk programs are needed. Genie [14] has been proposed to synthesize many possible user commands. Genie provides a template language for ThingTalk. From a small set of ***Primitive templates*** and ***Construct templates***, Genie can generate an exponential number of possible user commands so that virtual assistant developers can train the semantic parser.

### Our Contribution

The original Genie only supports user commands in English. This puts a restriction on non-English users. In this research, we extended Genie to support Chinese. Our contributions are as follows:

- The building of a Chinese dataset for all devices registered in Thingpedia;
- The design of ***Primitive*** and ***Construct templates*** of Chinese user commands for Genie;
- The building of a machine learning model that can convert Chinese user commands into ThingTalk programs.

To the best of our knowledge, this is the first Chinese compatible semantic parser based on Genie that enables user commands in Chinese to be accepted by Almond.

In the following sections, we introduce Almond and Genie in Section 2. We explain the details of Chinese ***Primitive*** and ***Construct templates*** in Section 3, and the machine learning models we tested are explained in Section 4. The experiment results are presented in Section 5. The conclusions and future works are given in Section 6.

## 2. Related Works

### 2.1. Almond, Thingpedia, and ThingTalk

Almond [12,13,14] is an open-source virtual assistant. It addresses issues such as *Privacy*, *Extensibility*, and *Programmability*. Almond stores all personal information and private data locally, such as user accounts and passwords for various Internet services and IoT devices. Almond can execute user commands represented as ThingTalk programs. ThingTalk is a high-level declarative domain-specific language designed to access Internet services and IoT devices, which are both referred to as skills in this article, via their Application Programming Interface (API) for ThingTalk. These APIs are registered in Thingpedia [15] by skill developers. Thingpedia is a skills library, or an application encyclopedia. When this article was written, it was an open API repository of 128 skills and 270 functions. It creates an interoperable web of skills. Service providers can add their own skills. They can either use built-in APIs to customize new skills, or upload packages of ThingTalk codes for their APIs registered in Thingpedia.

**Figure 1:**
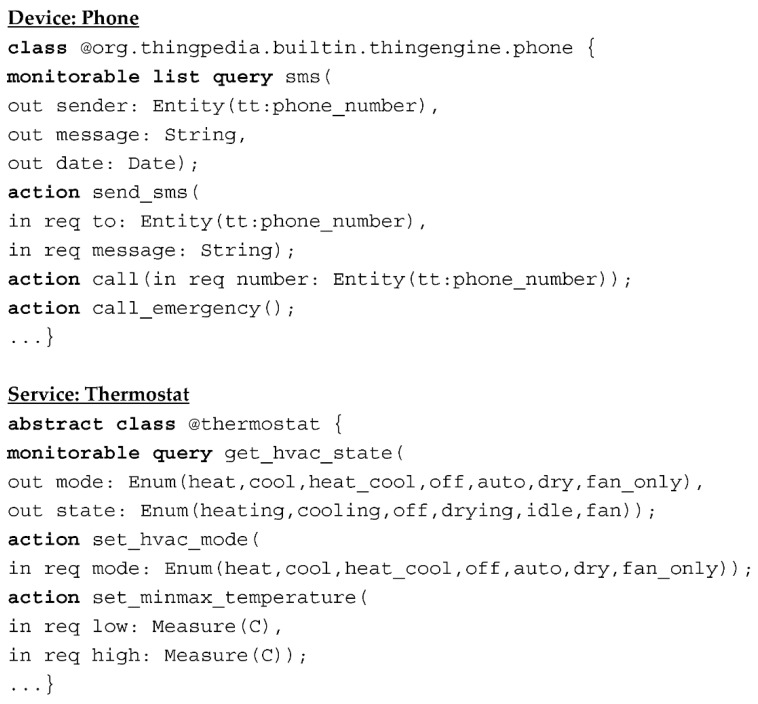
Snapshots of class examples in Thingpedia.

Figure 1 shows two class examples in Thingpedia. To be concise, we have omitted the grammar details for classes in Thingpedia. Classes in Thingpedia have two kinds of functions: ***query*** and ***action***. A ***query*** is a read-only operation. It returns results to users with no side effects. Some ***queries*** are ***monitorable***, which means the results can be monitored for changes. An ***action*** does not return results to users. It will change things, such as modifying users’ data. Data are passed into and out of the functions through named parameters, which can be required or optional.

Users can communicate with Almond in natural language. Almond uses a semantic parser to translate user commands in natural language into ThingTalk programs. Table 1 shows some command examples in English and their corresponding ThingTalk program. Again, we have omitted ThingTalk grammar details to be concise. ThingTalk is data focused and not control-flow focused. It has a single construct:

*s* [⇒ *q*]? ⇒ *a*; (1)

There are three types of clauses: ***stream*** (*s*), ***query*** (*q*), and ***action*** (*a*) clauses. The ***stream*** clause (*s*) determines when the rest of the program runs. It specifies the evaluation of the program as a continuous stream of events. It can be immediate or at a specified time, it can be a periodic timer, such as “once a week,” “two times per hours,” etc., or it can monitor the result of a ***monitorable* *query*** function defined in Thingpedia for changes. The optional ***query*** clause (*q*) specifies what data should be retrieved when the events occur. Query results can be optionally filtered with a boolean predicate. They can be used as an input parameter in a subsequent function invocation. The ***action*** clause (*a*) specifies what the program should do. It may show the data to the user or invoke an ***action*** function defined in Thingpedia. Table 2 shows some examples of user commands and their corresponding ThingTalk clause in relation to the three types, where placeholders for parameters are prefixed with $.

### 2.2. Semantic Parsing and Genie

Almond uses a semantic parser to translate user commands in natural language into ThingTalk programs. Specifically, semantic parsing here means the translation from natural language to a normal form, i.e., a machine understandable representation [16,17,18,19,20]. Zhong et al. designed a model for converting a natural language into a structure query language (SQL) [21]. They used reinforcement learning to train a sequence-to-sequence (seq2seq) model [22] consisting of a two-layer, bidirectional Long Short-Term Memory (LSTM) network [23]. We note that some SQL queries have no canonical forms, and the query equivalence is undecidable [24]. On the other hand, ThingTalk is designed to have a canonical form. User commands of the same semantic should be converted to the same ThingTalk program.

The semantic parser used by Almond is a very large artificial neural network (ANN). To train a large network, a huge amount of data are required. For example, for a better object classification, large image datasets such as ImageNet [25] have been built for the construction of machine learning models. It usually takes a lot of resources to collect and annotate such large datasets. The development of virtual assistants requires complex and expensive manual annotations by experts to gather enough training data as there are many different utterances of the same semantic. [26,27]. For an open-source project such as Almond, it is not easy to obtain training data at such a scale.

**Figure 2:**
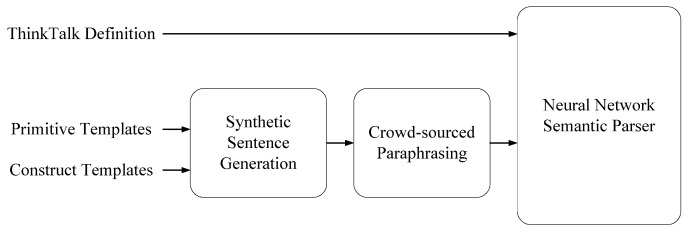
GENIE working flow.

To help virtual assistant developers acquire enough training data, Genie [14] was designed to synthesize a large amount of user commands in natural languages for large machine learning model training. Figure 2 shows the working flow of Genie. Genie takes advantage of natural language compositionality: an exponential number of possible user commands can be generated from a limited set of primitives. Based on the ThingTalk construct, Genie provides a template language so that users can generate synthetic user commands. It factors user command synthesis into ***primitive templates*** for skills, and ***construct templates*** for a natural language.

Skill designers should provide of a list of ***primitive templates***. Each ***primitive template*** consists of the code of a skill, an utterance describing it, and its grammar category. The syntax of ***primitive templates*** follows, where utterance *u*, belonging to category *cat*, maps to a ThingTalk clause, i.e., ***stream*** (*s*), ***query*** (*q*), or ***action*** (*a*). The utterance *u* may include placeholders, each of which is prefixed with $ and used as a parameter *pn* of type *t* in ThingTalk code.

*cat*:= *u* → *λ*([*pn*: *t*]∗) → [*s*|*q*|*a*] (2)

Table 3 shows some examples of ***primitive templates***. VP, NP, and WP refer to three grammar categories: verb phrase, noun phrase, and when phrase, respectively.

Skills designers should also provide a set of ***construct templates***, and to some extent rules of sentences, to map natural language compositional constructs to formal language operators. A ***construct template*** has the following form:

*lhs*:= [literal|*vn*: *rhs*]+ → *sf* (3)

It specifies that the derivation of a non-terminal category *lhs* can be constructed by combining the literals and variables *vn* of non-terminal category *rhs*, and then the semantic function *sf* is applied to compute the formal language representation. For example, the following ***construct templates*** describe a When-Do user command, “when something happens, do something.”

COMMAND:= *s*: WP ‘,’ *a*: VP → **return** *s* ⇒ *a*; COMMAND:= *a*: VP *s*: WP → **return** *s* ⇒ *a*;

When the following ***primitive templates*** are applied,

WP:= ‘when I post on facebook’ → monitor (@com.facebook.list_posts()) VP:= ‘notify me’ → notify                     

Genie would generate the following two When-Do commands,

“when I post on facebook, notify me.”

“notify me when I post on facebook.”

Both map to the following ThingTalk program:

monitor (@com.facebook.list_posts()) ⇒ notify

Please refer to Genie for more detail.

## 3. Chinese Compatible Genie

For Chinese users, Almond needs a semantic parser that can translate user commands in Chinese into ThingTalk programs. To train such a parser, a large amount of training data are required, i.e., pairs of user commands in Chinese and their corresponding ThingTalk programs. However, original Genie is designed for English.

To extend Genie for Chinese, based on the template approach described above, we carefully built ***primitive templates*** in Chinese for skills registered in Thingpedia, and ***construct templates*** for Chinese.

User command examples in English for skills registered in Thingpedia are first translated into Chinese and then segmented carefully into atomic elements in terms of their semantics. Proper sets of utterances of every atomic element are made up. In ***primitive template*** design, it is important to choose a suitable category so that any utterance can naturally be used as a component in a user command, as well as have a corresponding ThingTalk clause. In general, noun phrases (NP) are chosen for the ***query*** clause, verb phrases (VP) for the ***action*** clause, and when phrases (WP) for the ***stream*** clause. Table 4 shows some examples of ***primitive templates*** in Chinese designed for Genie toolkit. The utterance in each line is a translation from the corresponding utterance in Table 3.

We built a Chinese ***primitive template*** dataset for all 107 skills devices and 261 functions registered in Thingpedia. There were 649 Chinese ***primitive templates*** in the dataset.

Once the ***primitive templates*** have been built, the grammar and structures for possible user commands in Chinese for skills registered in Thingpedia are further analyzed, similar to the When-Do ***construct template*** examples in English as shown above.

There are four types of ***construct templates***, which are aggregation, timer, filters, constants, and parameters passing.

***Construct templates*** for aggregation operations, such as the sum, maximum, minimum, and average of the data, correspond to the specific calculation of the data retrieved from ***query*** commands. It is similar to the concept of operations on tables in a SQL database.

There are monitor, timer, and edge monitors for the ***stream*** clause in ThingTalk, such as “當 $x 更新時 (when $x is updated)” for the monitor, “每星期一次 (twice a week)” for the timer, and “當 $a 超過 $b (when $a is larger than $b)” for the edge monitor. For example, for an utterance such as “每天*t*點 (at *t* o’clock everyday),” the following timer ***construct templates*** are:

每天 *t*: *constant_time* 點 => *Timer* (*t*)

Users might use only a partial amount of data retrieved from a ***query*** clause. For example, users might want a list of emails that are labelled with a star. ThingTalk supports this filter in many different forms. For example, for an utterance such as “若*p*有*x* (if *p* has *x*)” or “若*p*包含*x* (if *p* contains *x*),” the following ***construct templates*** are:

*p*: *the_out_param_Array_Any* (“有” | “*包含*”) *x*: *constant_Any* => *makeFilter* (*p*, “*contains*”, *x*)

Taking this case as an example “當我收到信時 (when I got a letter), 回覆給寄件者 (reply to the sender).” This sentence can be segmented into two parts. The first part can be mapped to a ThingTalk ***query*** clause, *monitor* (*@com.gmail.inbox*()). The second part can be mapped to a ThingTalk ***action*** clause, *@com.gmail.reply* (*email_id* = *p_email_id*). As a result, the following ThingTalk program is:

*monitor* (*@com.gmail.inbox*()) => (*p_email_id*: *EmailAddress*) => *@com.gmail.reply* (*email_id* = *p_email_id*)

For all skills registered in Thingpedia, 292 ***construct templates*** in total were designed for Chinese.

## 4. Chinese Semantic Parser

To enable Chinese in Almond, two machine learning models were considered to be the semantic parser. One was a sequence-to-sequence (seq2seq) model [22], and the other was a Multiple Question Answering Network (MQAN) [28]. The sequence-to-sequence model is a state-of-the-art method, while MQAN has shown its outstanding performance in many natural language processing tasks.

### 4.1. Sequence to Sequence

The Deep Neural Network (DNN) is an extremely powerful machine learning model and can achieves many amazing performances in relation to difficult tasks such as object recognition [29,30] and speech recognition [31]. However, many DNNs can only be applied to problems whose inputs and outputs are of a fixed dimension.

Seq2seq [22] was initially designed for machine translation. It turns one sequence into another sequence. The main structure of the seq2seq model is based on LSTM architecture, which maps a variable length input sequence into a fixed length, fixed-dimension vector representation. It has been shown that LSTM can successfully learn long range temporal dependencies from data, i.e., the context in the utterance. In seq2seq, there one encoder maps each item into a corresponding vector, and one decoder maps the vector into an output item in reverse. Seq2seq has been applied successfully in language translation, text summarization, and even advanced mathematics, such as in the symbolic integration and resolution of differential equations [32].

In this work, we also applied a copy mechanism [33], which is based on attention mechanism [34,35], to help pass the parameters extracted from user commands in Chinese to the ThingTalk program.

### 4.2. Multiple Question Answer Network

**Figure 3:**
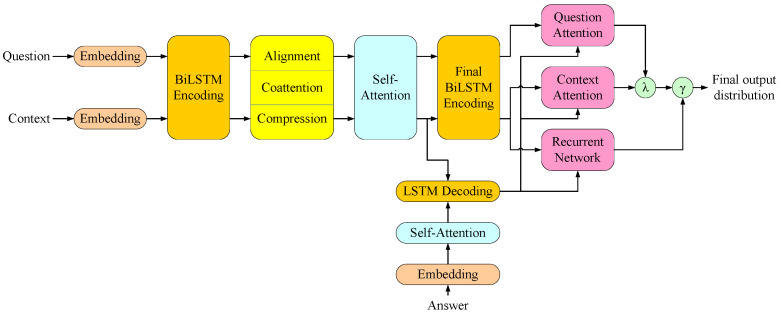
MQAN structure.

In Natural Language Decathlon (decaNLP), the use of MQAN (as shown in Figure 3) is generalized to solve ten different natural language processing tasks [28]. All tasks are framed as a question answering problem, with a question, a context, and an answer. For Almond, the translation from the user command into the ThingTalk program performed by a semantic parser can be converted to a question answering.

MQAN utilizes the attention mechanism in its structure, which helps the model focus on those corresponding parts in the original natural language command. This mechanism helps the semantic parser extract the important information from the user command and passes it to the target ThingTalk program. MQAN makes use of a novel dual co-attention and multi-pointer-generator decoder.

In MQAN, both the encoder and decoder use a deep stack of recurrent, attentive, and feed forward layers to learn the input representation. In the encoder, the question and context are first embedded by concatenating word and character embeddings, then they are fed into the encoder to construct the context and question representation. The decoder uses a mixed pointer-generator architecture to predict the target program. One token is generated each time.

Some question answering models assume that the answer can be copied from the context [36,37]. However, this is not always the case. Most of the tokens in the ThingTalk program cannot be chosen from the input natural language sentence. MQAN allows itself to select answer tokens from another word dictionary. The model can choose to generate next answer tokens from the embedded question, the context, or the word dictionary containing words that are not in the question or context.

## 5. Experiments

In this section, we present the results of the experiment.

For the 107 skills registered in Thingpedia, including IoT devices and web services, 649 Chinese ***primitive templates*** were built, and 292 ***construct templates*** for Chinese were designed.

### 5.1. Evaluation of Synthetic Sentences

We randomly chose 300 sentences which were synthetized by the Chinese-compatible Genie for web services such as Gmail, Spotify, Twitter, Facebook, Slack, and Imgur, and IoT devices such as a cell phone, television, bulb, blue-tooth speaker, and Fitbit. Those sentences were examined and paraphrased by experts.

There were 649 different ThingTalk programs and 2025 corresponding Chinese sentences in our dataset. We generated 2,301,938 synthetic sentences and corresponding ThingTalk programs using our ***primitive*** and ***construct templates*** for Chinese.

### 5.2. Sequenceto-Sequence Model

The sequence-to-sequence model was implemented using Tensorflow [38] and the Tensor2Tensor library [39]. We used the Stanford CoreNLP library [40] for preprocessing and Chinese word segmentation and name entity recognition. The words in the input Chinese utterances are embedded using fastText [41].

The structure of our model was a 2-layer LSTM with a hidden size of 128. We used dropout layers [42] between the two LSTM layers and an Adam [43] optimizer to update the model’s parameters.

We evaluated the performance of our models in three metrics, which were exact match, BLEU [44], and F1 token accuracy.

Exact match considers the result to be correct only if the output is an executable program with correct functions, parameters, and filters corresponding to the natural language command inputs. BLEU is a popular evaluation metric in many natural language processing tasks. We used it to evaluate the token level accuracy. The F1 token score considers both the precision (*P*) and recall (*R*) of the test to compute the score. *P* is the number of correct output tokens divided by the number of all output tokens. *R* is the number of correct output tokens divided by the number of all correct output tokens, which were the target ThingTalk programs in this case. The results of our sequence-to-sequence model can be found in Table 5.

### 5.3. Model

The MQAN model, implemented in PyTorch [45], was provided by decaNLP [28]. It is an open-source library. Again, we used the Stanford CoreNLP library [40] for preprocessing and Chinese word segmentation and name entity recognition. The words in the input Chinese utterances were embedded using fastText [41]. Words that do not have corresponding embeddings were assigned as zeros vectors. The 100-dimensional character n-gram embeddings were then concatenated to the fastText embeddings.

MQAN uses two self-attention and multi-head decoder attention layers. We used the transformer for our attention layers combined with a dropout layer on inputs to LSTM layers, following the co-attention and decoder layers. The model was trained using an Adam optimizer, with token-level cross-entropy loss.

We evaluated the performance of this model using the same three metrics, which were exact match, BLEU, and F1 token accuracy. The results can be found in Table 6.

### 5.4. Discussion

MQAN performed better than the sequence-to-sequence model. The structure was better at capturing the semantics in the natural sentence. We checked the synthetic sentences and found some reasons that could harm the performance of the semantic parser.

Although the sentences generated followed the rule we designed according to our construct templates, there were some sentences that did not make sense. For example, “get me a cup of latte when I receive a mail.” This sentence followed the when-do rule defined in our ***construct templates***, but we do not say this kind of sentence in real life. There were quite a few of these sentences in the synthetic sentences.

Before the sentences were fed into the semantic parser, we needed to first perform word segmentation. This is different from English because we do not have spaces between words in Chinese. The segmentation result could lead to some problems because sentences that have the same meaning with different nouns may cause the segmentation result of other words that are the same in the sentences to be different. In such a case, the model would treat those words as different tokens, which could lead to a bad performance. For example, “給我我的信 (give me my letters)” and “給我我的信件 (give me my letters)” have the same meaning; however, the word segmentations “給 我 我 的 信”, “給我 我 的 信件” are different. We removed those sentences that did not make sense in the training dataset and added more paraphrase sentences to enhance our model.

## 6. Conclusions

We extended Genie for Chinese, made the generating data process and training model available for Chinese users, and made the Almond virtual assistant available for Chinese users. We also built the first Chinese primitive templates for Genie and the first Genie semantic parser for Chinese, which is currently the best model. All of our codes have been merged into the original Genie project, which can be found in the Genie Github page. This work was an open-source project, and Chinese users could develop their devices and service based on our works. Our MQAN model achieved an exact match accuracy of 0.70, which is currently the best model.

Crowd-sourcing paraphrasing is important for the improvement of the Genie semantic parser. To make our Chinese semantic parser better, we need more training data from different distributions, including paraphrasing synthetic sentences and collecting real user cases. We believe by including paraphrasing sentences and users’ data, the performance of our Chinese semantic parser can be improved.

In addition to MQAN, many new neural network architectures have been proposed in recent years. For example, BERT [46] and GPT-3 [47] have performed very well in many significant natural language processing problems. We are currently working on the use of these new architectures for semantic parsing and ThingTalk code generation.

Today, it is easy to see an IoT scenario comprising several collaborative IoT networks; meanwhile each IoT network has also evolved in many other IoT scenarios. A new framework, MIoT (Multiple IoT) [48,49], has been proposed to treat these kinds of scenarios using social network analysis approaches. Social IoT (SIoT) [50] is an excellent attempt in this direction to explore the growing complexity in the collaboration of multiple IoT networks. Currently, we are also considering the semantics of information exchange between multiple IoT networks. ***Construct templates*** across multiple skills and the corresponding ThingTalk programs are under investigation.

## Figures and Tables

**Table 1:** User command examples in English and their corresponding ThingTalk program.

User Command	ThingTalk Program
Call the ambulance	now => @org.thingpedia.builtin.thingengine.phone. call_emergency()
Turn off the heater	now => @thermostat.set_hvac_mode param:mode:Enum(heat, cool, heat_cool, off) = enum:off
Turn on the air conditioner	now => @thermostat.set_hvac_mode param:mode:Enum(heat, cool, heat_cool, off) = enum:cool
Post a picture on Facebook	now => @com.facebook.post_picture()
Notify me when my location changes	monitor (@org.thingpedia.builtin.thingengine.phone. get_gps()) => notify
Notify me when I receive a text	monitor (@org.thingpedia.builtin.thingengine.phone. sms()) => notify

**Table 2:** ThingTalk clause examples.

Fragment of User Command	ThingTalk Clause Type	ThingTalk Clause
texts I received today sms I received today sms from today	query	(@org.thingpedia.builtin.thingengine. phone.sms()), date >= start_of(day)
when I receive a sms from ${p_sender} when I get at text from ${p_sender} when ${p_sender} sends me a text when ${p_sender} texts me when ${p_sender} sms me when ${p_sender} sends me an sms]	stream	monitor ( (@org.thingpedia.builtin.thingengine. phone.sms()), sender == p_sender)
call ${p_number} make a call to ${p_number} dial ${p_number}	action	@org.thingpedia.builtin.thingengine. phone.call(number = p_number)
turn the heating off turn off the heater turn off the ac switch off the heater switch the aircon off	action	@thermostat.set_hvac_mode (mode = enum(off))
set the temperature on my thermostat to ${p_value} set my thermostat to ${p_value} set the temperature to ${p_value} on my thermostat	action	@thermostat.set_target_temperature (value = p_value)

**Table 3:** Utterances in English and their ThingTalk clause examples.

Utterance	Cat	ThingTalk Clause
My photos on Facebook	NP	@com.facebook.list_photos()
My posts on Facebook	NP	@com.facebook.list_posts()
When I post on Facebook	WP	monitor @com.facebook.list_posts()
Post a picture on Facebook	VP	@com.facebook.post_picture()
Post $x on Facebook	VP	@con.facebook.post($x)

**Table 4:** Utterances in Chinese and their ThingTalk clause examples.

Utterance *	Cat	ThingTalk Clause
我臉書上的照片	NP	@com.facebook.list_photos()
我的臉書貼文	NP	@com.facebook.list_posts()
當我貼文的時候	WP	monitor @com.facebook.list_posts()
貼圖到臉書上	VP	@com.facebook.post_picture()
po $x 到臉書	VP	@con.facebook.post($x)

* The utterances are the translation of Table 3. 臉書 refers to Facebook.

**Table 5:** Experiment results of the Seq2seq model.

	Exact Match	BLEU	F1 Token Accuracy
Seq2seq	0.44	0.73	0.83
Seq2seq + fastText	0.46	0.75	0.85

**Table 6:** Experiment results of the MQAN model.

	Exact Match	BLEU	F1 Token Accuracy
MQAN	0.70	0.82	0.86
MQAN+fastText	0.70	0.82	0.88

## Data Availability

Not acceptable.
